# Common Variants in a Novel Gene, *FONG* on Chromosome 2q33.1 Confer Risk of Osteoporosis in Japanese

**DOI:** 10.1371/journal.pone.0019641

**Published:** 2011-05-06

**Authors:** Ikuyo Kou, Atsushi Takahashi, Tomohiko Urano, Naoshi Fukui, Hideki Ito, Kouichi Ozaki, Toshihiro Tanaka, Takayuki Hosoi, Masataka Shiraki, Satoshi Inoue, Yusuke Nakamura, Naoyuki Kamatani, Michiaki Kubo, Seijiro Mori, Shiro Ikegawa

**Affiliations:** 1 Laboratory for Bone and Joint Diseases, Center for Genomic Medicine, RIKEN, Tokyo, Japan; 2 Laboratory for Statistical Analysis, Center for Genomic Medicine, RIKEN, Tokyo, Japan; 3 Department of Geriatric Medicine, Graduate School of Medicine, The University of Tokyo, Tokyo, Japan; 4 Department of Anti-Aging Medicine, Graduate School of Medicine, The University of Tokyo, Tokyo, Japan; 5 Division of Gene Regulation and Signal Transduction, Research Center for Genomic Medicine, Saitama Medical University, Hidaka, Japan; 6 Department of Pathomechanisms, Clinical Research Center for Rheumatology and Allergy, National Hospital Organization Sagamihara National Hospital, Sagamihara, Japan; 7 Department of Internal Medicine, Tokyo Metropolitan Geriatric Hospital, Tokyo, Japan; 8 Laboratory for Cardiovascular Diseases, Center for Genomic Medicine, RIKEN, Yokohama, Japan; 9 Department of Advanced Medicine, National Center for Geriatrics and Gerontology, Obu, Japan; 10 Research Institute and Practice for Involutional Diseases, Azumino, Japan; 11 Human Genome Center, Institute of Medical Science, The University of Tokyo, Tokyo, Japan; 12 Laboratory for International Alliance, Center for Genomic Medicine, RIKEN, Yokohama, Japan; 13 Laboratory for Genotyping Development, Center for Genomic Medicine, RIKEN, Yokohama, Japan; Pennington Biomedical Research Center, United States of America

## Abstract

Osteoporosis is a common disease characterized by low bone mass, decreased bone quality and increased predisposition to fracture. Genetic factors have been implicated in its etiology; however, the specific genes related to susceptibility to osteoporosis are not entirely known. To detect susceptibility genes for osteoporosis, we conducted a genome-wide association study in Japanese using ∼270,000 SNPs in 1,747 subjects (190 cases and 1,557 controls) followed by multiple levels of replication of the association using a total of ∼5,000 subjects (2,092 cases and 3,114 controls). Through these staged association studies followed by resequencing and linkage disequilibrium mapping, we identified a single nucleotide polymorphism (SNP), rs7605378 associated with osteoporosis. (combined *P* = 1.51×10^−8^, odds ratio = 1.25). This SNP is in a previously unknown gene on chromosome 2q33.1, *FONG*. *FONG* is predicted to encode a 147 amino-acid protein with a formiminotransferase domain in its N-terminal (FTCD_N domain) and is ubiquitously expressed in various tissues including bone. Our findings would give a new insight into osteoporosis etiology and pathogenesis.

## Introduction

Osteoporosis (MIM166710) is one of the most common skeletal diseases affecting more than 200 million individuals in the world. Its prevalence is estimated to be increasing dramatically as population ages [1]. Osteoporosis is characterized clinically by reduced bone mass and compromised bone strength, leading to an increased risk of fracture.

Osteoporosis is a polygenic disease; Both environmental and genetic factors contribute to its etiology and pathogenesis [2]. To understand its genetic factor, identification of its susceptibility gene(s) is important. There are several experimental approaches to identify susceptibility genes for osteoporosis. One is a candidate gene approach. Genes relevant to bone metabolism and disease genes of rare monogenic bone diseases are widely studied by this approach and the association with osteoporosis has been reported in many genes; however, only a few genes like those for estrogen receptor 1 (*ESR1*), α1 chain of type I collagen (*COL1A1*) and low-density lipoprotein 5 (*LRP5*) are replicated for their association [3]–[5], including large-scale meta-analyses using different ethnic populations [6], [7].

Another approach is a genome-wide association study (GWAS). GWAS has a great power to detect genetic variants with less than moderate effects [8], [9]. Its notable advantage is a potential for finding previously unknown susceptibility genes [10]. Recently, several groups conducted GWAS and identified many loci associated with susceptibility to osteoporosis mainly in Caucasian [11]–[16]; however, the genetic contribution to osteoporosis is not entirely known.

To uncover additional susceptibility gene(s) for osteoporosis, we conducted a GWAS in Japanese followed by staged replication studies. We found a SNP (rs7605378) on chromosome 2q33.1 that showed significant association (*P* = 1.51×10^−8^) with susceptibility to osteoporosis. The SNP is in a previously unknown gene, which we named *FONG*.

## Results

### GWAS

**Figure 1:**
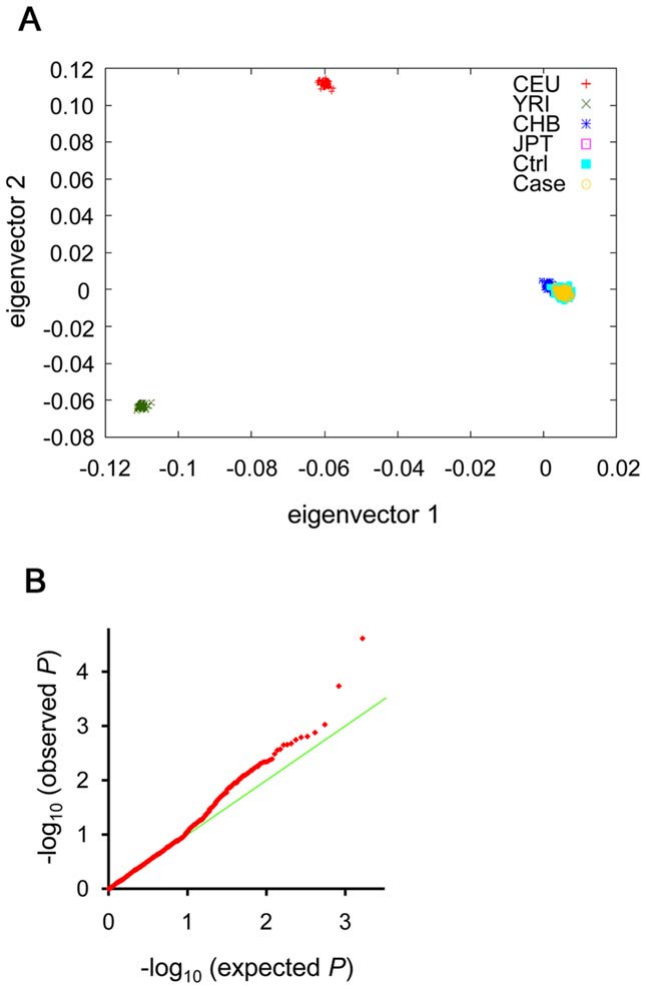
Evaluation of population stratification for the GWAS. (A) Principal component analysis. Samples in the GWAS and in HapMap database are analyzed by a program, Smartpca [20], and plotted for the first (X axis) and the second (Y axis) principal components (PCs), respectively. Our case and control samples are plotted in a single cluster of Japanese. (B) Quantile-quantile (Q-Q) plots of allelic association using Fisher's exact (allelic) test in Discovery 2. Under the null hypothesis of no association at any locus, the points would be expected to follow the slope line (light green). Deviations of the points (red dots) from the line correspond to loci that deviate from the null hypothesis. The genetic inflation factor lambda is 1.04.

To identify the causal SNPs associated with osteoporosis, we used staged association method [17], [18] (Fig. S1). As the first stage of discovery (Discovery 1), we performed GWAS and genotyped 268,064 SNPs that covered 56% of common SNPs in Japanese, in 190 cases and in 1,557 controls registered in the BioBank Japan (BBJ) [19]. After passing through the quality control (QC) filter described in the Material and Method, we successfully obtained genotyping data for 224,507 SNPs. The χ^2^ distributions for the association tests across the tested SNPs showed a low possibility of overall systematic bias (genomic inflation factor: λ_GC_ = 1.02). We further performed a principal component analysis (PCA) [20] for the samples and found no evidence for population stratification (Fig. 1A).

### SNPs reported by previous GWASs

We checked our GWAS data for 94 SNPs in 45 genes reported in previous GWAS on osteoporosis [6], [12], [14]–[16], [21]–[24]. Twelve SNPs in eight genes were included in our platform, successfully genotyped and passed the QC filter (Table S1). Five SNPs among them showed *P* values below 0.05. Three SNPs in the *PLCL1* gene [12] showed significant association after the Bonferroni correction (*P*<4.17×10^−3^ = 0.05/12).

### Step-wise screening

As the second stage of discovery (Discovery 2), we selected 3,000 SNPs showing the smallest *P* values in Discovery 1 and genotyped these SNPs in an independent set of subjects composed by 526 cases and 1,537 controls. We successfully obtained genotyping data for 1,654 SNPs. Quantile-quantile plots revealed the presence of a number of SNPs associated with osteoporosis (Fig. 1B). The χ^2^ distributions for the association tests across the tested SNPs showed a low possibility of false positive association due to population stratification (λ_GC_ = 1.04).

After the Discovery stages, no SNP exceeded the genome-wide significance threshold. We therefore selected three SNPs that showed the smallest *P* values (*P*<1.0×10^−3^ in Discovery 2) for the replication. In the discovery stages, there were age and sex differences between the cases and controls. Therefore, to exclude the false positive due to the differences, we used age- and sex-adjusted cases and controls in the replication stages (Table S2). As the first stage of replication (Replication 1), we genotyped the SNPs in an independent set of female subjects composed by 1,326 cases and 1,292 controls. We set significance threshold in this stage after the Bonferroni correction for multiple testing to *P*<1.67×10^−2^ ( = 0.05/3). Only one SNP, rs7605378 on chromosome 2q33.1 showed significance (*P* = 2.99×10^−3^) (Table 1). To validate the association of rs7605378, we further genotyped it in an independent female population of 240 cases and 285 controls as the second stage of replication (Replication 2), and found further replication of the significant association (*P* = 3.97×10^−2^) (Table 1).

**Table 1:** Association of rs7605378 with osteoporosis.

Population	Number of subject		RAF		*P* value^a^	OR (95% CI)^b^	*P*_het_ c
	Case	Control	Case	Control			
Discovery 1	190	1557	0.647	0.556	7.11×10^−4^	1.46 (1.17–1.83)	
Discovery 2	523	1537	0.599	0.542	1.16×10^−3^	1.27 (1.10–1.46)	
Replication 1	1326	1292	0.564	0.524	2.99×10^−3^	1.18 (1.06–1.31)	
Replication 2	240	285	0.600	0.537	3.97×10^−2^	1.29 (1.01–1.65)	
*All combined* d	2279	4671			1.51×10^−8^	1.25 (1.16–1.35)	0.37

RAF: risk allele frequency, OR: odds ratio, CI: confidence interval.

a *P* values are calculated using the Pearson's χ^2^ test for the allele model.

b OR of the risk allele from the two-by-two allele frequency table.

c Heterogeneity is calculated using the Mantel-Haenszel method.

d The combined *P* value of the four studies (Discovery 1, 2 and Replication 1, 2) is calculated using the Mantel-Haenszel method.

Thus, through the staged association study using independent populations, we identified and validated the association of rs7605378, a new susceptibility loci for osteoporosis. The combined *P* value was 1.51×10^−8^ (OR = 1.25; 95% CI: 1.16–1.35) (Table 1).

### LD mapping

**Figure 2:**
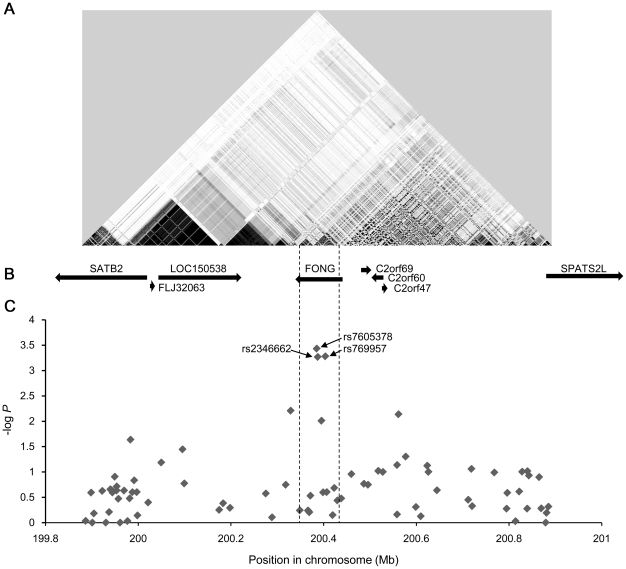
Association signals around rs7605378 on chromosome 2 in the GWAS stage. (A) LD plot for the studied region based on the *r* ^2^ statistic. The intensity of shading is proportional to *r* ^2^. (B) Genomic structure around the *FONG* region. (C) Results of GWAS for osteoporosis in a Japanese population. The log10-transformed *P* values are plotted on the y axis.

To define the linkage disequilibrium (LD) block containing rs7605378, we examine SNPs around rs7605378 (Fig. 2). We referenced the International HapMap Project database (release 23a) and selected SNPs that had *D*' value of >0.8 to rs7605378 and a minor allele frequency of >0.1. The LD block around rs7605378 contained 51 HapMap SNPs and one hypothetical gene, *LOC348751*. Next, we selected tag SNPs including rs7605378 that covered all 51 SNPs with an *r* ^2^ value of >0.8. After genotyping the 14 tag SNPs for 2,042 cases (Discovery 1, 2 and Replication 1) and 1,292 controls (Replication 1), we found no more significantly associated SNP than rs7605378 (Table 2). Then, we analyzed haplotype association using the 14 tag SNPs for the LD block. We did not find any haplotypes that had more significant association than rs7605378 (Table 3).

**Table 2:** Association of 14 selected tag SNPs for the rs7605378 region with osteoporosis.

SNP	Number of subject		MAF		*P* value^a^	OR (95% CI)^b^
	Case	Control	Case	Control		
rs12373788	2036	1290	0.131	0.137	4.70×10^−1^	1.05 (0.91–1.22)
rs7572473	2039	1292	0.314	0.276	9.54×10^−4^	0.83 (0.75–0.93)
rs12615435	2039	1289	0.222	0.201	3.96×10^−2^	0.88 (0.78–0.99)
rs10931875	2038	1290	0.300	0.296	7.49×10^−1^	0.98 (0.88–1.09)
rs12473679	2038	1290	0.481	0.438	6.59×10^−4^	0.84 (0.76–0.93)
rs6759644	2039	1290	0.076	0.095	6.04×10^−3^	1.28 (1.07–1.52)
rs17529497	2038	1292	0.280	0.318	7.70×10^−4^	1.20 (1.08–1.34)
rs6743271	2034	1289	0.442	0.417	4.42×10^−2^	0.90 (0.82–1.00)
rs4673491	2037	1290	0.083	0.074	1.50×10^−1^	0.87 (0.73–1.05)
rs7605378	2038	1292	0.419	0.476	3.96×10^−6^	1.26 (1.14–1.39)
rs2030653	2036	1290	0.218	0.204	1.75×10^−1^	0.92 (0.81–1.04)
rs2164889	2039	1292	0.207	0.190	9.22×10^−2^	0.90 (0.79–1.02)
rs12470986	2039	1289	0.183	0.158	9.14×10^−3^	0.83 (0.73–0.96)
rs10203122	2038	1290	0.389	0.347	4.84×10^−4^	0.83 (0.75–0.92)

MAF: minor allele frequency, OR: odds ratio, CI: confidence interval.

a *P* values are calculated using the Pearson's χ^2^ test for the allele model.

b ORs are for the major allele versus the minor allele.

**Table 3:** Haplotype association analysis using 14 tag SNPs in the LD block containing rs7605378.

Haplotype*	Frequency		*P* value	OR (95% CI)
	Case	Control		
22222212212222	0.241	0.274	6.17×10^−3^	1.19 (1.05–1.35)
22111221222121	0.176	0.163	2.29×10^−1^	0.91 (0.79–1.06)
21221221222211	0.150	0.122	3.33×10^−3^	0.79 (0.67–0.93)
21222222221222	0.096	0.105	3.26×10^−1^	1.10 (0.91–1.31)
12222222121222	0.063	0.053	1.35×10^−1^	0.84 (0.66–1.06)
22222222212222	0.050	0.053	6.62×10^−1^	1.06 (0.83–1.35)
12211121212222	0.045	0.058	3.33×10^−2^	1.30 (1.02–1.67)
22211121212222	0.029	0.032	4.97×10^−1^	1.11 (0.81–1.52)
22222212221211	0.018	0.018	9.40×10^−1^	0.98 (0.65–1.48)
22111222212222	0.016	0.012	2.44×10^−1^	0.75 (0.47–1.20)
21221222212222	0.015	0.009	7.59×10^−2^	0.64 (0.39–1.08)
21221212212222	0.011	0.005	1.55×10^−2^	0.46 (0.24–0.88)

All haplotypes with a frequency of >1% in the osteoporosis population of set 3 (case 3, control 3) are shown.

1 and 2 indicate the minor and major allele in the population, respectively.

OR: odds ratio, CI: confidence interval.

*The alleles of 14 SNPs (from left to right, rs12373788, rs7572473, rs12615435, rs10931875, rs12473679, rs6759644, rs17529497, rs6743271, rs4673491, rs7605378, rs2030653, rs2164889, rs12470986 and rs10203122) are shown.

### Identification of *FONG*

**Figure 3:**
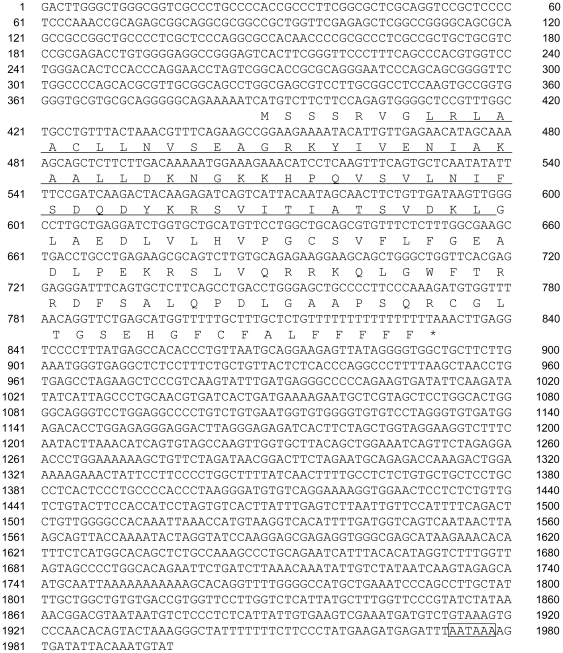
Nucleotide and deduced amino acid sequences of *FONG*. A domain homologous to the FTCD_N domain is underlined. A stop codon is indicated by an asterisk, and the putative poly-A addition signal is enclosed in an open box. Multiple transcription start sites (TSSs) were identified by 5′-RACE, but only the major TSS is shown.

In the NCBI genome database (build 36.3), rs7605378 lay within a hypothetical gene, *LOC348751*. Because the *LOC348751* transcript was based on *in silico* predictions and expressed sequence tags (ESTs) only, we tried to clone a full sequence of the actually expressed transcript from bone by RACE and RT-PCR. We identified a new transcript that overlapped with, but was different from *LOC348751*. The longest transcript was 1,997 bp in length with a predicted protein of 147 amino acids (Fig. 3). A protein motif analysis program (http://www.ebi.ac.uk/Tools/InterProScan/) predicted that this protein contained a signal peptide and a formiminotransferase domain in its N-terminal (FTCD_N domain). We named this newly identified gene *FONG* (for formiminotransferase N-terminal sub-domain containing gene: AB568489). *FONG* had many alternative splicing variants and multiple transcription start sites (TSSs). We performed luciferase assays in two human osteoblastic cell lines, MG-63 and SaOS-2, and confirmed that the region containing 500-bp upstream of the major TSS and 5′UTR possessed promoter activity (data not shown).

### Expression of *FONG*

**Figure 4:**
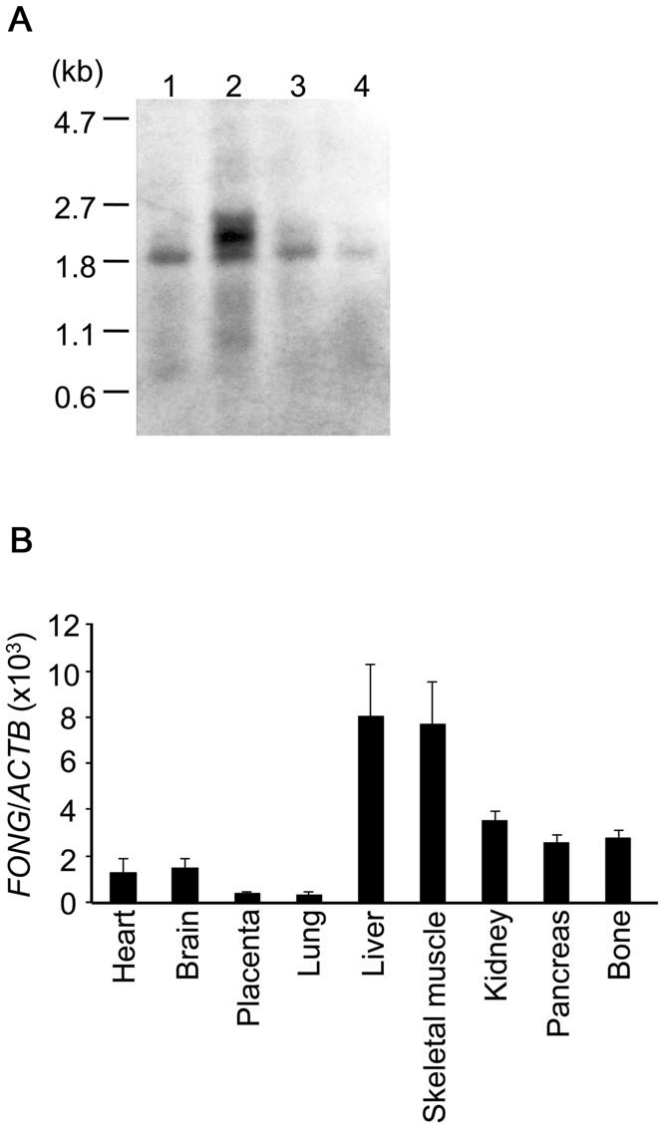
Tissue expression of *FONG*. (A) Northern blotting in human tissues. Lane 1, kidney; lane 2, skeletal muscle; lane 3, liver; lane 4, bone. (B) Quantitative real-time PCR in various human tissues. *FONG* is highly expressed in liver, skeletal muscle, and moderately expressed in bone. Data represent the mean ratios of *FONG* mRNA to β-actin (*ACTB*) mRNA ± s.e.m. of two independent experiments.

To confirm the expression and size of the *FONG* transcript, we carried out Northern analysis and identified two transcripts, approximately 2.2 kb and 2.0 kb in length. The 2.0-kb transcript was common to all tissues examined (Fig. 4A). We also examined *FONG* expression in various human tissues using real-time PCR. *FONG* was ubiquitously expressed in various tissues including bone (Fig. 4B).

### Determination of the most associated SNP

To locate the functional, osteoporosis-associated SNP, we searched for SNPs in ∼25 kb region that had *r* ^2^>0.8 with rs7605378 by direct sequencing of genomic DNA from 24 case subjects. We found 20 previously unknown SNPs in addition to 39 known SNPs in the HapMap database (Table S3). After calculating pairwise *r* ^2^ values for all 59 SNPs in this region, we selected 22 tag SNPs with *r* ^2^>0.95. In addition to the six SNPs already genotyped, we selected 16 additional SNPs and genotyped them for 697 cases (a part of Replication 1) and 567 controls (a part of Replication 1). Two SNPs (SNP11 and rs58319901) showed more significant association than rs7605378 (Table S4). We genotyped them in additional samples consisting of 2,042 cases (Discovery 1, 2 and Replication 1) and 1,292 controls (Replication 1); however, both SNP did not show more significant association than rs7605378 (Table S5). In the ∼25 kb region that had *r* ^2^>0.8 with rs7605378, we found 12 SNPs were in perfect LD (*r* ^2^ = 1) with rs7605378 (Table S3). Because they were all in perfect LD with each other, we could not pinpoint a candidate causal SNP among these 13 SNPs.

## Discussion

We have used a staged association design that provides multiple levels of replication followed by resequencing of the LD block, and identified a SNP, rs7605378 that is associated with susceptibility to osteoporosis. Only a few osteoporosis GWAS have been reported in Asian, which have identified a few specific genes like *JAG1* and *ALDH7A1* [16], [24]. This study represents the first GWAS of osteoporosis in Japanese. In the discovery stages (Discovery 1 and 2), there were age and sex differences between cases and controls. However, the allele frequency of rs7605378 in the stages was not significantly different between males and females (*P* = 0.33). We re-evaluated the association of rs7605378 in the stages by adjusted sex (female-only analysis). Because of the decreased number of samples, the combined *P* value of rs7605378 in the discovery stages became a little high, but their ORs are similar after adjustment (with males: 1.32; female only: 1.34). In addition, we adjusted the age of the female samples by a logistic regression and found no significant change in the ORs. Therefore, we considered age and sex difference between cases and controls did not confound the association. rs7605378 exceeds definite genome-wide significance level even after Bonferroni correction, which is known to be very conservative. The SNP is in a previous unknown gene, *FONG*. To our knowledge, this is the first report of a novel gene as a susceptibility gene of osteoporosis.

Comparison of our GWAS data with SNPs identified in previous GWASs on osteoporosis [6], [12], [14]–[16], [21]–[24] showed five SNPs in three genes (*PLCL1*, *DOK6*, and *MEF2C*) with *P* values below 0.05 (Table S1). These results in Japanese, a different ethnic population from the previous studies would support their association. We could not deny the association of other promising SNPs identified in previous GWASs, because our GWAS has a limited power to detect association due to the relatively small sample size and moderate coverage of the genome. Ethnic difference in genetic background may also preclude replication of these SNPs; some SNPs identified in previous GWAS in Caucasians are found monomorphic in Japanese in the present study. Three SNPs in *PLCL1* showed significant association even after correction of the multiple testing in our study (Table S1). However, whether these results really support the association of *PLCL1* is not clear, because their allele frequencies had not been disclosed in the previous study [12] and hence the direction of the association of these SNPs (i.e., which alleles were the susceptibility alleles) remains unknown.

In the current public databases, rs7605378 was located in *LOC348751*, a hypothetical gene based on *in silico* prediction. In the prediction, *LOC348751* consists of 5 exons; however, our RT-PCR experiments could only prove a part of exon 2 and exons 3–5. The predicted exon 1 was not present. Therefore, we performed the 5′- and 3′-RACE using bone cDNA and determined the actual mRNA sequence and the gene structure. We found that *FONG* consisted of 4 exons; the exon 1 and a part of exon 2 of *LOC348751* were not present (Fig. S2). In addition, *FONG* had many splicing variants. Most of the variants contained exons 2 and 3 of *FONG* in common, but the first and last exons had variants. The new transcript that we have found (Fig. 3) existed in several tissues like kidney, skeletal muscle, liver and bone (Fig. 4A) and its predicted protein sequence was conserved among several species. Therefore, we think the transcript that we have found (Fig. 3) is a major splicing variant of *FONG*.

The N-terminal amino acid sequences of *FONG* corresponding to the FTCD-N domain are highly conserved from *Xenopus* to human, suggesting its important biological role. FTCD is a mammalian metabolic enzyme which involves in conversion of histidine to glutamic acid, and the FTCD-N domain has a transferase activity that transfers a formimino group from N-formimino-L-glutamic acid to tetrahydrofolate to generate glutamic acid and 5-formiminotetrahydrofolate [25]. The glutamate signaling is considered to play an important role in bone homeostasis. For instance, L-glutamic acid is known to be secreted by osteoclasts and knockout mice of the glutamate transporter 1 develop osteoporosis [26]. These lines of evidence suggest that *FONG* have a potential to regulate bone metabolism.

While preparing this paper, annotation of *LOC348751* in public database is updated and *LOC348751* has come to be described as a miscRNA. However, the length of the *LOC348751* mRNA in the database (NR_034096.1) is shorter than that of the *FONG* mRNA that we experimentally determined. The new *LOC348751* mRNA consisted of 966 bp and its open reading frame (ORF) encoded only 77 amino acid residues, while the *FONG* mRNA consisted of 1,997 bp and its ORF encoded 147 amino acids. Besides, the protein sequence of *FONG* is well conserved between different species. We suspect that *LOC348751* is one of the *FONG* variant transcripts. However, because we have not yet succeeded in proving the existence of the FONG protein experimentally, we cannot deny the possibility that *FONG* functions as a miscRNA. Recently, many miscRNAs are found and their important roles in pathogenesis of diseases have been known [27]–[29].

The most associated SNP, rs7605378, is in perfect LD with 12 SNPs (Table S3). All of them are in the *FONG* region, but they do not cause amino acid substitutions. These SNPs are located in intron 3 or 3′ flanking region. Therefore, they may have affect *FONG* expression. Two ESTs containing rs7605378 are reported. In our experiments, we could not find any *FONG* splicing variant(s) containing these ESTs. However, some splicing variants seems tissue specific and *FONG* may have other splicing valiant(s). Further analysis of these transcripts may provide a new insight into *FONG* function.

In conclusion, this study identified a previous unknown gene, *FONG* as a novel susceptibility gene for osteoporosis. Although *FONG* function and its osteoporosis-causing mechanism are largely unknown, our findings would provide a new insight into the complex genetic architecture of osteoporosis. The identified variants are warranted by further biological and clinical investigation.

## Materials and Methods

### Subjects

We carried out a stepwise case-control association method as previously described [10], [30]–[32], using several independent populations (Table S2). Case and control subjects used in discovery stages were obtained from the BBJ [19]. Osteoporosis was diagnosed according to the criteria of Japanese Osteoporosis Society as bone mineral density (BMD) being <70% of young adult mean (YAM) at either the lumbar spine or femoral neck [33]. This criteria is equivalent to that of the World Health Organization (WHO) of T-score<−2.5. BMD at the lumbar spine (L2-4 or L1-4) and/or femoral neck was measured by dual energy radiograph absorptiometry with standard protocols. All individuals in the osteoporosis populations were postmenopausal and/or over 60 years female. The controls were the subjects with various diseases other than osteoporosis as previously described [19]. For the replication study, the criteria of cases are same as the discovery stage and that of controls are postmenopausal females and/or females over 60 years. The cases in Replication 1 were also obtained from BBJ. The cases in Replication 2 and the controls in Replication 1 and 2 were obtained from unrelated ambulatory volunteers. All the participants provided written informed consent. This research project was approved by the ethical committees at Institute of Medical Science, the University of Tokyo and Center for Genomic Medicine, RIKEN.

### SNP genotyping

Using standard protocols, genomic DNA was extracted from peripheral blood leukocytes. In Discovery 1, 268,064 SNPs from autosomal chromosomes were genotyped by using high-density oligonucleotide arrays (Perlegen Sciences). These SNPs were selected from JSNP [34] or HapMap database [35] as tagging SNPs for Japanese. SNPs having call rate >90% and no significant deviation from Hardy-Weinberg equilibrium (HWE; P≥1.0×10^−6^) were used for the analysis of association. A total of 224,507 SNPs were passed QC filters and were further analyzed for their association. Among the SNPs analyzed in Discovery 1, top 3,000 SNPs showing the smallest *P* values were selected for Discovery 2. Genotyping of Discovery 2 was conducted using the multiplex-PCR invader assay [36] or high-density oligonucleotide arrays (Perlegen Sciences). In this stage, 1,654 SNPs passed QC filters (call rate of ≥0.9, *P* value of HWE≥0.01 in controls, and concordance rates of >90% between Perlegen and Invader assays using randomly selected 94 case samples and 752 control samples). Among the SNPs analyzed in the discovery stages, top three SNPs showing the smallest *P* values were selected for the replication study in the replication stage. Genotyping in the stage was conducted using the multiplex-PCR invader assay or the TaqMan assay (Applied Biosystems). All cluster plots were checked by visual inspection and SNPs with ambiguous calls were excluded.

### Statistical analysis

In the discovery stage, Fisher's exact test was applied to two-by-two contingency table in three genetic models: an allele frequency model, a dominant-effect model, and a recessive-effect model. At the replication stage, the association was assessed using χ^2^ test that was applied to two-by-two contingency table in the three genetic models. Odds ratios and confidence intervals were calculated using the minor allele as a reference. The haplotype association was analyzed using Haploview software [37]. A PCA was conducted to detect population stratification [20]. A combined *P* value and heterogeneity were calculated using the Mantel-Haenszel method.

### RACE, RT-PCR and real-time PCR

5′- and 3′- RACE were performed using Marathon-Ready cDNAs for human kidney, skeletal muscle and liver (Clontech). A human bone cDNA library was constructed using FastTrack 2.0 mRNA Isolation kit (Invitrogen) and SMART RACE cDNA amplification kit (Clontech) according to the manufacture's protocol. A bone cDNA was synthesized using Multiscribe reverse transcriptase and a random hexamer primer (Applied Biosystems). The bone cDNA and multiple tissue cDNA panels (Clontech) were used for PCR experiments to examine tissue-specific expression of *FONG*. Quantitative real-time PCR was carried out using an ABI PRISM 7700 sequence detector with Quantitect SYBR Green PCR Kit (Qiagen) in accordance with the manufacturers' instructions.

### Northern blotting

The cDNA fragment corresponding to nucleotides 413–731 of *FONG* was cloned into the pCR2.1 TOPO vector (Invitrogen). The DIG-labeled probe was synthesized from the constructed vector using DIG RNA Labeling Kit (Roche). Total RNAs of kidney, skeletal muscle and liver were purchased from Clontech (The skeletal muscle: seven male/female Caucasians, kidney: 14 male/female Caucasians, liver: a male Caucasian). Total RNAs of bone was extracted from bone tissues of nine male/female Japanese. mRNAs were synthesized using FastTrack 2.0 mRNA Isolation kit (Invitrogen) and 2 µg of mRNAs were used for gel electrophoresis. Transfer, hybridization and detection were done using DIG Easy Hyb and DIG Wash and Block Buffer set (Roche) according to the manufacturer's instructions.

## Supporting Information

Figure S1 **Design of our staged association study.** We performed a genome-wide screening as the first stage of discovery (Discovery 1), followed by further examination of the top findings (Discovery 2). We then performed two replications (Replication 1 and 2) and resequencing of the LD block. In each stage, we consider the minimum *P* value in three genetic models.
(TIF)

Figure S2 **A schematic diagram of the gene structures of** ***FONG*** **and** ***LOC348751*** **.** Boxes indicated exons. All exon-intron junctions conformed to the “ag-gt” rule. The open boxes represent the untranslated regions and the closed boxes the coding regions. The reading frames of *FONG* and *LOC348751* are different.
(TIF)

Table S1 **Comparison of the previous GWAS and the current GWAS.**
(XLS)

Table S2 **Basal characteristics of the subjects.**
(XLS)

Table S3 **SNPs in the same linkage disequilibrium block (** ***r*** **^2^>0.8) with rs7605378.**
(XLS)

Table S4 **Association of additional tag SNPs in** ***FONG*** **region with osteoporosis.**
(XLS)

Table S5 **Association of rs7605378, SNP11 and rs58319901 with osteoporosis.**
(XLS)

## References

[1] Reginster JY, Burlet N (2006). Osteoporosis: a still increasing prevalence.. Bone.

[2] Peacock M, Turner CH, Econs MJ, Foroud T (2002). Genetics of osteoporosis.. Endocr Rev.

[3] Liu YZ, Liu YJ, Recker RR, Deng HW (2003). Molecular studies of identification of genes for osteoporosis: the 2002 update.. J Endocrinol.

[4] Ralston SH, de Crombrugghe B (2006). Genetic regulation of bone mass and susceptibility to osteoporosis.. Genes Dev.

[5] Liu YJ, Shen H, Xiao P, Xiong DH, Li LH (2006). Molecular genetic studies of gene identification for osteoporosis: a 2004 update.. J Bone Miner Res.

[6] Rivadeneira F, Styrkarsdottir U, Estrada K, Halldorsson BV, Hsu YH (2009). Twenty bone-mineral-density loci identified by large-scale meta-analysis of genome-wide association studies.. Nat Genet.

[7] Mann V, Hobson EE, Li B, Stewart TL, Grant SF (2001). A COL1A1 Sp1 binding site polymorphism predisposes to osteoporotic fracture by affecting bone density and quality.. J Clin Invest.

[8] Easton DF, Pooley KA, Dunning AM, Pharoah PD, Thompson D (2007). Genome-wide association study identifies novel breast cancer susceptibility loci.. Nature.

[9] Consortium WTCC (2007). Genome-wide association study of 14,000 cases of seven common diseases and 3,000 shared controls.. Nature.

[10] Miyamoto Y, Shi D, Nakajima M, Ozaki K, Sudo A (2008). Common variants in DVWA on chromosome 3p24.3 are associated with susceptibility to knee osteoarthritis.. Nat Genet.

[11] Kiel DP, Demissie S, Dupuis J, Lunetta KL, Murabito JM (2007). Genome-wide association with bone mass and geometry in the Framingham Heart Study.. BMC Med Genet.

[12] Liu YZ, Wilson SG, Wang L, Liu XG, Guo YF (2008). Identification of PLCL1 gene for hip bone size variation in females in a genome-wide association study.. PLoS One.

[13] Richards JB, Rivadeneira F, Inouye M, Pastinen TM, Soranzo N (2008). Bone mineral density, osteoporosis, and osteoporotic fractures: a genome-wide association study.. Lancet.

[14] Styrkarsdottir U, Halldorsson BV, Gretarsdottir S, Gudbjartsson DF, Walters GB (2008). Multiple genetic loci for bone mineral density and fractures.. N Engl J Med.

[15] Xiong DH, Liu XG, Guo YF, Tan LJ, Wang L (2009). Genome-wide association and follow-up replication studies identified ADAMTS18 and TGFBR3 as bone mass candidate genes in different ethnic groups.. Am J Hum Genet.

[16] Kung AW, Xiao SM, Cherny S, Li GH, Gao Y (2010). Association of JAG1 with bone mineral density and osteoporotic fractures: a genome-wide association study and follow-up replication studies.. Am J Hum Genet.

[17] Nakashima M, Chung S, Takahashi A, Kamatani N, Kawaguchi T (2010). A genome-wide association study identifies four susceptibility loci for keloid in the Japanese population.. Nat Genet.

[18] Tomlinson IP, Webb E, Carvajal-Carmona L, Broderick P, Howarth K (2008). A genome-wide association study identifies colorectal cancer susceptibility loci on chromosomes 10p14 and 8q23.3.. Nat Genet.

[19] Nakamura Y (2007). The BioBank Japan Project.. Clin Adv Hematol Oncol.

[20] Price AL, Patterson NJ, Plenge RM, Weinblatt ME, Shadick NA (2006). Principal components analysis corrects for stratification in genome-wide association studies.. Nat Genet.

[21] Styrkarsdottir U, Halldorsson BV, Gretarsdottir S, Gudbjartsson DF, Walters GB (2009). New sequence variants associated with bone mineral density.. Nat Genet.

[22] Ahn J, Yu K, Stolzenberg-Solomon R, Simon KC, McCullough ML (2010). Genome-wide association study of circulating vitamin D levels.. Hum Mol Genet.

[23] Hsu YH, Zillikens MC, Wilson SG, Farber CR, Demissie S (2010). An integration of genome-wide association study and gene expression profiling to prioritize the discovery of novel susceptibility Loci for osteoporosis-related traits.. PLoS Genet.

[24] Guo Y, Tan LJ, Lei SF, Yang TL, Chen XD (2010). Genome-wide association study identifies ALDH7A1 as a novel susceptibility gene for osteoporosis.. PLoS Genet.

[25] Murley LL, MacKenzie RE (1995). The two monofunctional domains of octameric formiminotransferase-cyclodeaminase exist as dimers.. Biochemistry.

[26] Morimoto R, Uehara S, Yatsushiro S, Juge N, Hua Z (2006). Secretion of L-glutamate from osteoclasts through transcytosis.. EMBO J.

[27] Akhtar N, Rasheed Z, Ramamurthy S, Anbazhagan AN, Voss FR (2010). MicroRNA-27b regulates the expression of matrix metalloproteinase 13 in human osteoarthritis chondrocytes.. Arthritis Rheum.

[28] Li H, Xie H, Liu W, Hu R, Huang B (2009). A novel microRNA targeting HDAC5 regulates osteoblast differentiation in mice and contributes to primary osteoporosis in humans.. J Clin Invest.

[29] Miyaki S, Sato T, Inoue A, Otsuki S, Ito Y (2010). MicroRNA-140 plays dual roles in both cartilage development and homeostasis.. Genes Dev.

[30] Kubo M, Hata J, Ninomiya T, Matsuda K, Yonemoto K (2007). A nonsynonymous SNP in PRKCH (protein kinase C eta) increases the risk of cerebral infarction.. Nat Genet.

[31] Hata J, Matsuda K, Ninomiya T, Yonemoto K, Matsushita T (2007). Functional SNP in an Sp1-binding site of AGTRL1 gene is associated with susceptibility to brain infarction.. Hum Mol Genet.

[32] Ozaki K, Ohnishi Y, Iida A, Sekine A, Yamada R (2002). Functional SNPs in the lymphotoxin-alpha gene that are associated with susceptibility to myocardial infarction.. Nat Genet.

[33] Orimo H, Hayashi Y, Fukunaga M, Sone T, Fujiwara S (2001). Diagnostic criteria for primary osteoporosis: year 2000 revision.. J Bone Miner Metab.

[34] Haga H, Yamada R, Ohnishi Y, Nakamura Y, Tanaka T (2002). Gene-based SNP discovery as part of the Japanese Millennium Genome Project: identification of 190,562 genetic variations in the human genome. Single-nucleotide polymorphism.. J Hum Genet.

[35] Frazer KA, Ballinger DG, Cox DR, Hinds DA, Stuve LL (2007). A second generation human haplotype map of over 3.1 million SNPs.. Nature.

[36] Ohnishi Y, Tanaka T, Ozaki K, Yamada R, Suzuki H (2001). A high-throughput SNP typing system for genome-wide association studies.. J Hum Genet.

[37] Barrett JC, Fry B, Maller J, Daly MJ (2005). Haploview: analysis and visualization of LD and haplotype maps.. Bioinformatics.

