# Supplementary figures and images for: Common Variants in a Novel Gene, FONG on Chromosome 2q33.1 Confer Risk of Osteoporosis in Japanese
Source: PLoS One. 2011 May 6;6(5):e19641. doi: 10.1371/journal.pone.0019641 (PMC3089633; doi:10.1371/journal.pone.0019641)

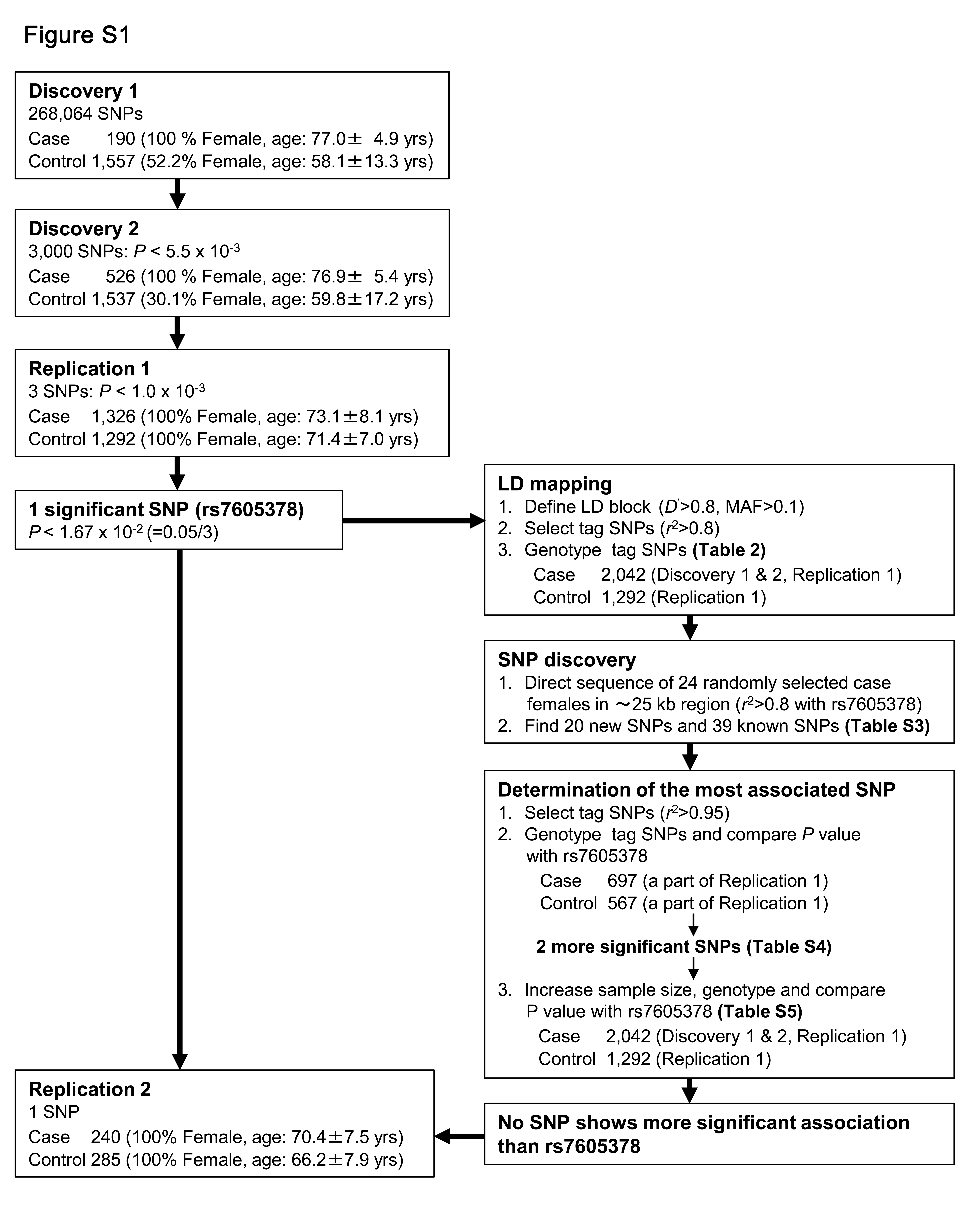

Supplement: Figure S1 — Design of our staged association study. We performed a genome-wide screening as the first stage of discovery (Discovery 1), followed by further examination of the top findings (Discovery 2). We then performed two replications (Replication 1 and 2) and resequencing of the LD block. In each stage, we consider the minimum P value in three genetic models. (TIF) [file pone.0019641.s001.tif]

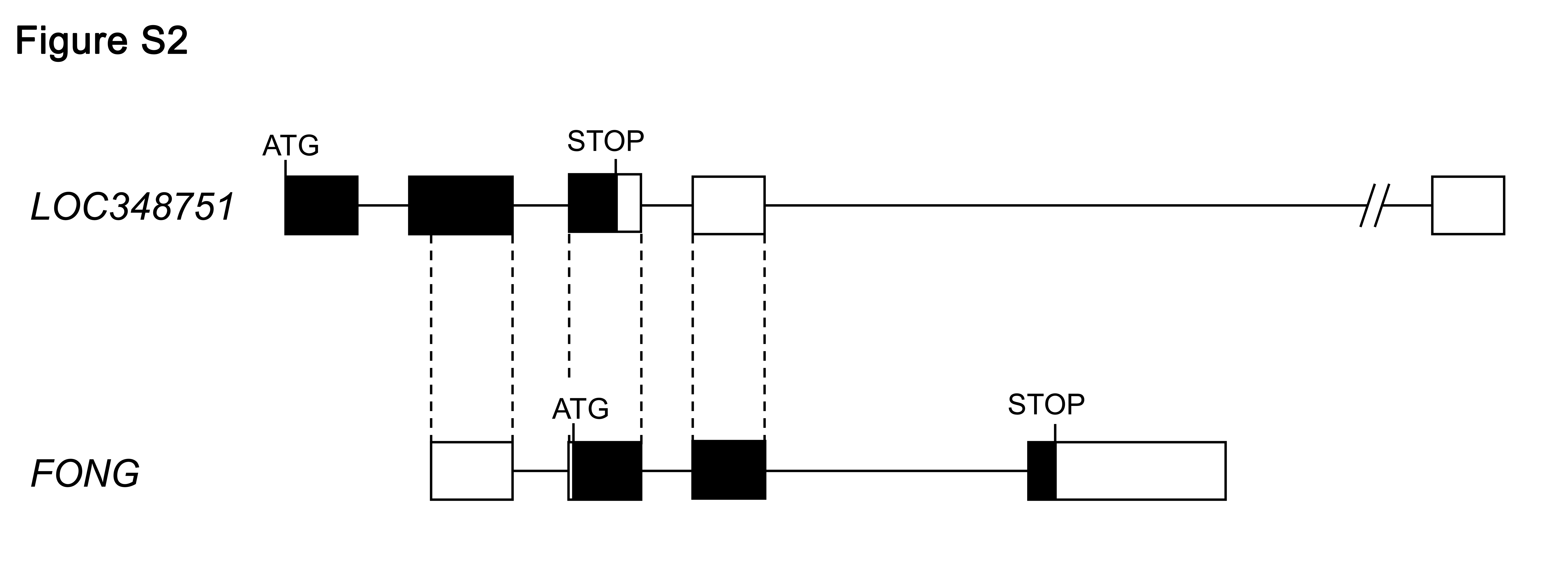

Supplement: Figure S2 — A schematic diagram of the gene structures of FONG and LOC348751 . Boxes indicated exons. All exon-intron junctions conformed to the “ag-gt” rule. The open boxes represent the untranslated regions and the closed boxes the coding regions. The reading frames of FONG and LOC348751 are different. (TIF) [file pone.0019641.s002.tif]
